# Supplementary material for: High Antibiotic Resistance of Helicobacter pylori and Its Associated Novel Gene Mutations among the Mongolian Population
Source: Microorganisms. 2020 Jul 16;8(7):1062. doi: 10.3390/microorganisms8071062 (PMC7409119; doi:10.3390/microorganisms8071062)
Supplement: Supplementary file 1 [file microorganisms-08-01062-s001.pdf]

Supplementary Material

Table 1. Levofloxacin resistant strains variants

No.	GyrA protein variants	Number of Resistant Strains	
		with variant	without variant
1	S5L	12	13
2	V6I	8	17
3	**N87K**	6	19
4	**A88P**	1	24
5	**D91N/Y/G**	15	10
6	D143E	4	21
7	D161N	3	22
8	M191I	20	5
9	A197V	1	24
10	V199I	6	19
11	G208E	7	18
12	E211K	1	24
13	A231V	1	4
14	A283V	1	24
15	H354R	22	3
16	E394G	1	24
17	A379V	1	24
18	R397Q	9	16
19	I381V	1	24
20	Q403P	3	22
21	A407V	3	22
22	M413I	2	23
23	L452F/I	2	23
24	D454A/S/T	18	7
25	D455N	1	24
26	D464Y/E	2	23
27	G468E	14	11
28	L474F	1	24
29	S482P	1	24
30	P484Q	11	14
31	R486C	1	24
32	S492A/V	17	8
33	N495S	2	23
34	A524V	4	21
35	K527R	18	7

36	L536T	1	24
37	S539N	17	8
38	H569R	1	24
39	A578S	1	24
40	I587V	8	17
41	A594D/T	9	16
42	P595S	2	23
43	D610N	1	24
44	N620K	3	22
45	E632G	21	4
46	I639V	1	24
47	V643I	5	21
48	R635FS	1	24
49	S636FS	1	24
50	C637S	1	24
51	I639M	1	24
52	V655I	17	8
53	N659D	4	21
54	H622Y	2	23
55	H668Y	15	10
56	I671L	3	22
57	P676S	5	20
58	E679D	24	1
59	I683M	12	13
60	T686N/S	14	11
61	T687A	12	13
62	I691R	7	18
63	K694R	15	10
64	S708N	6	19
65	D709G	2	23
66	G711S	1	24
67	G733E	23	2
68	V741I/M	19	6
69	L746I	1	24
70	G755S	11	14
71	L766F	2	23
72	R784K	13	12
73	A790T	10	15
74	N797D	11	14

75	Asn789_Ala790delinsLysThr	1	24
76	V802I	1	24
77	M803V	11	14
78	V805A	11	14
79	P814S	1	24
80	L817S	2	23
81	T819N/I	13	12
82	S820P	3	22
83	S821P/Q	8	17
84	A822T	3	22
85	Q823P/L	5	20
86	N824L	20	5
87	L825F	20	5
88	F826E	20	5
